# Bacterial and archaeal community distributions and cosmopolitanism across physicochemically diverse hot springs

**DOI:** 10.1038/s43705-023-00291-z

**Published:** 2023-08-18

**Authors:** Chanenath Sriaporn, Kathleen A. Campbell, Martin J. Van Kranendonk, Kim M. Handley

**Affiliations:** 1 School of Biological Sciences, The University of Auckland, Auckland, New Zealand; 2 School of Environment & Te Ao Mārama – Centre for Fundamental Inquiry, The University of Auckland, Auckland, New Zealand; 3 Australian Centre for Astrobiology, School of Biological, Earth and Environmental Sciences, University of New South Wales, Sydney, Australia

**Keywords:** Environmental microbiology, Microbial ecology

## Abstract

Terrestrial hot springs harbor diverse microbial communities whose compositions are shaped by the wide-ranging physico-chemistries of individual springs. The effect of enormous physico-chemical differences on bacterial and archaeal distributions and population structures is little understood. We therefore analysed the prevalence and relative abundance of bacteria and archaea in the sediments (*n* = 76) of hot spring features, in the Taupō Volcanic Zone (New Zealand), spanning large differences in major anion water chemistry, pH (2.0–7.5), and temperature (17.5–92.9 °C). Community composition, based on 16S rRNA amplicon sequence variants (ASVs) was strongly influenced by both temperature and pH. However, certain lineages characterized diverse hot springs. At the domain level, bacteria and archaea shared broadly equivalent community abundances across physico-chemically diverse springs, despite slightly lower bacteria-to-archaea ratios and microbial 16S rRNA gene concentrations at higher temperatures. Communities were almost exclusively dominated by Proteobacteria, Euryarchaeota or Crenarchaeota. Eight archaeal and bacterial ASVs from Thermoplasmatales, Desulfurellaceae, Mesoaciditogaceae and Acidithiobacillaceae were unusually prevalent (present in 57.9–84.2% of samples) and abundant (1.7–12.0% sample relative abundance), and together comprised 44% of overall community abundance. Metagenomic analyses generated multiple populations associated with dominant ASVs, and showed characteristic traits of each lineage for sulfur, nitrogen and hydrogen metabolism. Differences in metabolic gene composition and genome-specific metabolism delineated populations from relatives. Genome coverage calculations showed that populations associated with each lineage were distributed across a physicochemically broad range of hot springs. Results imply that certain bacterial and archaeal lineages harbor different population structures and metabolic potentials for colonizing diverse hot spring environments.

## Introduction

Globally, the environmental factor found to affect microbial community composition the most is salinity [1]. However, at smaller geographical scales or within single biomes where salinity is stable (e.g., freshwater environments), other physicochemical factors, such as temperature and pH become significant [2]. These factors are expected to have an even greater effect in hot springs, where temperature and pH ranges are extreme compared to many other environments, e.g., pH ranges of −0.8 to 10.5 and temperature ranges of <10 to >100°C [3–6]. The extremes of these ranges are almost at the boundaries of conditions able to support life [7–11], and are inhabited by acidophilic, alkaliphilic, and (hyper)thermophilic microorganisms [6, 12–15].

Phylogenetically diverse microorganisms are found across the physico-chemical ranges of hot springs. For example, the bacterial phyla Proteobacteria and Aquificae are common inhabitants of various hot springs such as Taupō Volcanic Zone (TVZ, New Zealand), Yellowstone National Park (YNP, USA), and Hveragerði (Iceland) [6, 12, 15–17]. In addition, various archaea from Euryarchaeota (e.g., *Thermoplasma*) and Crenarchaeota (e.g., *Sulfolobus*) have been isolated or molecularly characterized from hot springs globally [18, 19]. Likewise, several thermophilic cyanobacterial genera, including *Synechococcus*, *Leptolyngbya*, and *Calothrix*, have been reported across alkaline hot springs worldwide [20]. While both hot spring bacteria and archaea have been determined via 16S rRNA characterization [12, 21–23], most of these studies utilized different primer sets for bacteria and archaea, making archaeal and bacterial relative abundances incomparable. Moreover, of the studies that have addressed both domains together using a single universal prokaryotic primer set [6, 24], some were potentially affected by known primer biases against archaea [25], suggesting that further research is needed to understand the relative contributions of archaea and bacteria in various hot spring settings [6].

High phylogenetic diversity among hot spring microorganisms may be expected given the distinct adaptations required to survive across large ranges in temperature and pH [26, 27]. For example, genome streamlining is a characteristic of microorganisms adapted to high temperatures, allowing low costs of energy maintenance and increased fitness [28]. Thermophiles may also possess higher concentrations of saturated lipids compared to non-thermophiles to increase their membrane integrity [29]. To maintain pH homeostasis of their intracellular regions in highly acidic environments, acidophiles possess mechanisms to pump out excess intracellular protons, whereas alkaliphiles increase proton influx into cells to maintain intracellular charge balance [30, 31]. Accordingly, previous studies have shown that temperature and pH are strong drivers of differences in diversity and composition among hot spring microbial communities [6, 12–15]. Despite the selective pressures exerted by extreme physicochemical differences among hot springs, some groups of bacteria and archaea in hot spring communities, such as *Acidithiobacillus*, *Venevibrio* and some Thermoplasmatales, are present in hot springs spanning large physicochemical ranges [6, 15]. For example, we previously observed the same cosmopolitan, genome-streamlined, *Acidithiobacillus* species (particularly the TVZ_G3 group) in hot spring sediments spanning temperatures of 17.5–92.9°C and pHs of 1.0–7.5 [17]. Nevertheless, the cosmopolitanism of other dominant hot spring microorganisms across varying hot spring physicochemistries is yet to be determined.

Here, we evaluated the relative abundances of bacteria and archaea, and their 16S rRNA gene copy numbers, across a wide range of hot spring physicochemistries (i.e., temperature, pH, and major anions), and assessed cosmopolitanism among dominant hot spring microorganisms. To do this, we sampled 76 subaqueous sediments from four geothermal areas (up to 65 km apart) across the TVZ, New Zealand. We then examined the composition of 16S rRNA gene amplicon sequence variants (ASVs), and the abundance and prevalence of microbial ASVs/variants. The relationship of dominant and prevalent variants – those with >1% relative abundance in the overall microbial community and >50% prevalence across samples – to temperature and pH was determined. In addition, using metagenomics, genomes of abundant and prevalent variants were examined to evaluate population structures underpinning cosmopolitanism and the traits of these populations related to sulfur, nitrogen and hydrogen metabolism. Results provide insights into phylogenetic distributions, cosmopolitanism and niche differentiation among hot spring adapted microorganisms.

## Materials and methods

### Sample collection and physicochemical measurements

Seventy-six sediment samples were collected from four geothermal areas in the TVZ located 1–65 km apart – Parariki thermal stream, the Sinter Flats lagoon area at the Rotokawa geothermal field, Tikitere geothermal field, and Waiotapu Scenic Reserve  located 1–65 km apart, in February and November 2019 (Table S1; Fig. 1 from [15]). Across these geothermal areas, eight sites with multiple co-located hot spring features, including 38 hot spring vents, 27 hot spring outflows, and 11 geothermally-influenced streams were sampled. Samples included 3–5 spatial replicates (<0.5 m apart) per feature, except for at Waiotapu A, which comprised a complex of numerous small vents with replicated chemistries, and Waiotapu B, where its vent and outflow were relatively small. Sediment was collected from a few millimeters to centimeters below the water surface into sterile 50 mL centrifuge tubes, transported on dry ice, and stored at -80°C. The pHs and temperatures of the hot spring fluids were measured *in situ* using a WTW 330i handheld meter (WTW GmbH, Germany) (Table S1). Major water anion data for Parariki stream, Rotokawa, and Tikitere were derived from prior studies [32–34], and Waiotapu water chemistry data were from the 1000springs project (http://1000springs.org.nz).

### DNA extraction, amplicon sequencing and quantification, and metagenomic sequencing

DNA extractions, 16S rRNA gene V4-V5 amplicon sequencing from all 76 sediment samples, and metagenome sequencing from 18 samples, along with genome assembly, binning and annotation were undertaken as previously described [17] and are summarized in Supplementary Information. Droplet Digital PCR (ddPCR) was used to quantify the concentration of 16S rRNA genes in each sample using the same primers (without Illumina adapters) and PCR conditions with additional signal stabilizing steps [15].

### Amplicon data analyses

QIIME2 (version 2019.10) was used to process demultiplexed sequence reads by read joining, quality filtering (Q score cutoff of 25), and denoising (with singletons removed) [35]. Tables of OTUs (clustered at 99% identity) and ASVs (sequences 100% identical) were generated using VSEARCH and deblur plug-ins, respectively [36, 37]. Taxonomy was assigned using the SILVA database version 132 [38] and q2-feature-classifier plug-in [39]. Rarefaction curves were generated using R (version 4.0.2) with the R package vegan (version 2.5-6). Alpha and beta-diversity were determined using vegan and visualized using ggplot2 (version 3.3.2). Statistical correlations were generated using R package ggpubr (version 0.4.0). Correlations and statistical significances of correlations were determined using Pearson’s correlation coefficients and t-distribution tables (df = *n*–1), respectively.

### Metagenomic data analyses

Metagenome-assembled genomes (MAGs) shared across up to 18 sediment sample assemblies were grouped by 98% and 99% similarity threshold using dRep version 1.4.3 [40] and are referred to as equivalent populations. Barrnap version 0.9 [41] was used to extract 16S rRNA genes from MAGs. FastANI version 1.33 was used to calculate pairwise average nucleotide identities (ANI) [42]. DRAM version 1.4.6 was used for annotation against KEGG-based KOfam, UniRef and Pfam databases downloaded 26-May-2023 [43–46]. Single-nucleotide polymorphisms (SNPs) detection was analyzed using Snippy version 4.6.0 [47]. For full metagenomic methodology, refer to Supplementary Information.

## Results and discussion

### Physicochemical diversity of studied hot spring settings

The four geothermal areas sampled differed based on fluid pH and major anions, ranging from acid-sulfate-chloride to acid-sulfate-bicarbonate [48, 49] (Table S1). Because temperature is a major controller of microbial composition and diversity in hot springs [6, 12, 15, 23, 50], we also sampled multiple features at each site, including vents for high temperatures (38.0–92.9 °C), outflows for moderate temperatures (17.5–69.1 °C), and geothermally-influenced streams for low temperatures (18.3–33.4 °C) (Figure S1). Site temperatures and pHs differed by up to 57.4 °C and 3.5 units, with overall temperatures and pHs being 17.5–92.9 °C (average 47.7 °C) and 2.0–7.5 (average 4.0) (Figure S1). In the TVZ, most hot springs are highly acidic, in contrast, for example, to the YNP, which includes more circumneutral pH springs [51].

### Bacteria and archaea were relatively abundant across acidic to circumneutral hot spring sediments

**Fig. 1:**
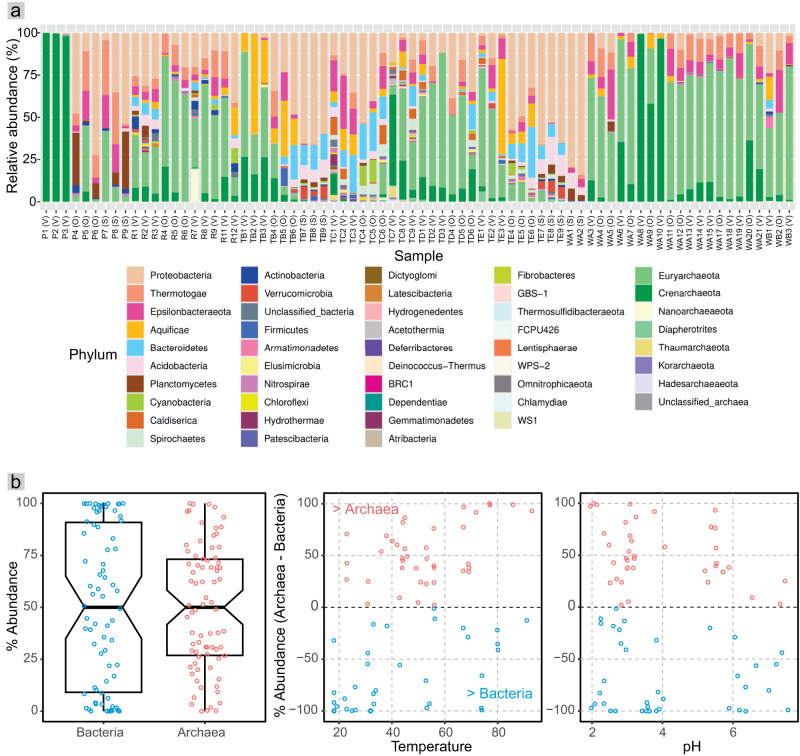
Distribution and relative abundances of bacteria and archaea in hot spring sediments of the Taupō Volcanic Zone (TVZ), New Zealand. **a** Relative abundance of phyla based on 16S rRNA gene amplicons for prokaryotic communities associated with hot spring sediments across all samples. Each sample contained between 24 and 2,046 ASVs (or 16 to 274 after rarefying to the minimum number of sequences in a sample, 673, Table S1). Symbols in parentheses after the sample names refer to the local hot spring feature sampled: V = Vent, O = Outflow, and S = Geothermally-influenced stream. **b** Plots showing the relative abundances of bacteria and archaea across all samples (left), and their percent difference across hot springs with varying temperatures (middle) and pHs (right). Percent difference was calculated as archaeal abundance per sample - bacterial abundance per sample. The gap between pH 4.1 and 5.3 reflects the lack of hot springs with these pHs in the TVZ.

A total of 23,225 ASVs, comprising 47 phyla including unclassified archaea and bacteria, were obtained based on 16S rRNA amplicon sequences from hot spring and hot spring-influenced sediments. Of these, 55.3% of the ASVs, in terms of richness, belonged to two archaeal phyla, Euryarchaeota and Crenarchaeota, and one bacterial phylum, Proteobacteria (Fig. 1a). These three phyla were also dominant, and together comprised 72.1% of the overall hot spring microbial community abundance. While specific hot spring communities tended to be dominated by either bacteria or archaea, overall, bacterial and archaeal abundances were roughly similar (56.3% and 43.7%, respectively) and both exhibited broadly similar distribution patterns with respect to temperature and pH (Fig. 1b). However, we observed significant and opposing correlations between temperature and the relative abundances of archaea (R = 0.42, *p* = 0.00014, Pearson’s correlation coefficient) and bacteria (R = −0.42, *p* = 0.00014) (Figure S2). No correlations were observed with pH. These trends are consistent with archaeal preferences for high-temperature niches and the early characterization of archaea solely as extremophiles [52]. Such associations are supported by archaeal adaptations conferring high-temperature tolerance, such as the presence of tetraether lipids in cell membranes [26]. Despite this, bacteria still comprised, on average, over 40% of communities from hot springs with temperatures over 70 °C (45.4% on average) and also over 80 °C (43.2% on average). In fact, some of the most well-studied (hyper)thermophiles from hot springs are bacteria (e.g., Aquificales and *Thermus* spp.) [53], and studies of other TVZ hot spring features (water and siliceous deposits) also showed that bacterial taxa, such as *Hydrogenobaculum* (Aquificae), *Venevibrio* (Aquificae), and *Acidithiobacillus* (Proteobacteria) were common in high-temperature springs [6, 15, 50].

Euryarchaeota and Crenarchaeota, which dominated the archaeal fraction of hot spring communities in this study and were major constituents of the combined bacterial and archaeal communities, comprise a high proportion of the archaeal communities of many hot springs globally [18, 22, 23, 54, 55]. Other commonly observed archaeal phyla in this study (Fig. 1a) are also common inhabitants of hot springs elsewhere. For example, Thaumarchaeota, Diapherotrites, Hadesarchaeaeota, Korarchaeota, and Nanoarchaeaeota have been reported from hot springs in Kamchatka, YNP, and Iceland [56–59]. Likewise, the dominant and commonly observed bacterial phyla detected in our study (e.g., Proteobacteria, Thermotogae, Epsilonbacteraeota, Aquificae, and Planctomycetes) are found in geographically diverse hot springs [12–14, 24]. Taken together, these data suggest widespread occurrences of the same bacterial and archaeal lineages across hot springs globally. While our results show that archaea are at least as abundant as bacteria in acidic to circumneutral pH TVZ hot spring sediments, an extensive study of hot spring water samples in the TVZ showed that the abundance of archaea (6.4%) was much lower than bacteria (93.6%) [6], either reflecting underestimation of archaea due to a V4 primer bias [6] (Supplementary Information), or substantial differences between water and sediment community compositions [24].

### Microbial composition, biomass, and diversity differed strongly with changes in temperature and/or pH

Beta-diversity analysis indicated that microbial community composition in hot spring and hot spring-associated sediments was shaped by temperature and pH (and major anions, which are pH-associated) (Figure S3), comparable to previous studies [6, 12–14, 24]. Higher temperature vent communities were differentiated from cooler outflow or geothermally-influenced stream communities in the constructed ordination. Similarly, greater diversity was previously reported at a distal area than at the spring vents [60]. Although the streams were cooler on average than hot spring outflows (26.4 ± 6.7°C versus 36.1 ± 14.1°C), half of the geothermally-influenced stream sediment communities were indistinguishable from outflow communities, reflecting their acidity and the large geothermal inputs received.

**Fig. 2:**
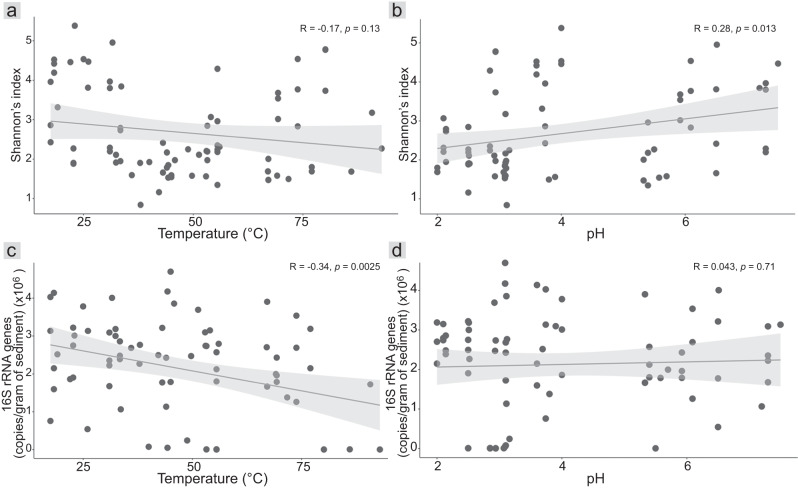
Hot spring community alpha diversity and 16S rRNA gene copy number relationships with physicochemistry. Scatter plots showing (**a**) a non-significant correlation between Shannon’s indices and temperature (°C) (R = −0.17, *p* = 0.13); (**b**) a significantly positive correlation between Shannon’s indices and pH (R = 0.28, *p* = 0.013); (**c**) a significantly negative correlation between 16S rRNA gene copies and temperature (°C) (R = −0.34, *p* = 0.0025); (**d**) a non-significant correlation between 16S rRNA gene copies and pH (R = 0.043, *p* = 0.71). Correlations and statistical significances of correlations were determined using Pearson’s correlation coefficients and t-distribution tables (df = n–1), respectively. Lines represent linear regressions and shaded areas represent 95% confidence intervals.

Biomass inferred from 16S rRNA gene concentrations was significantly and negatively correlated with temperature (R = −0.34, *p* = 0.0025; Fig. 2c), but not pH (R = 0.043, *p* = 0.71; Fig. 2d). Studies of creek sediments and soils have also reported a lack of correlation with pH [61, 62]. Correlated temperature and 16S rRNA gene concentrations across wide-ranging temperatures (17.5–92.9 °C) and eight sites, together with similar trends observed elsewhere, suggests inferred biomass is consistently reduced in higher temperature springs (e.g., 39.3–74.1°C across a single site [63]; 57–100 °C across three sites [12]). The trend identified here remained significantly negative after excluding geothermally-influenced streams (R = −0.3, *p* = 0.016; Figure S4). At high-temperature sites (≥70 °C), we detected almost two-fold lower copy number concentrations on average (1,329,131 ± 1,284,851 copies/gram of sediment) compared to low temperatures sites (≤40 °C, 2,571,607 ± 894,876 copies/gram of sediment). Moreover, at some extremely high-temperature sites (>80 °C), copy numbers were 1000-fold lower than at most low and moderate temperature hot spring sites (Figure S5a), highlighting the negative effect of temperature on inferred microbial biomass (Fig. 2c). Overall, concentration ranges were similar to those reported from hot springs elsewhere [12, 24]. However, copy numbers were 10 to 10,000-fold lower than those reported from freshwater and marine sediments [64, 65], potentially owing to the higher temperatures of hot springs in general. It is worth noting that although ddPCR quantifies gene copy numbers and not actual biomass or cell numbers, concentrations in this study were similar to those obtained using direct cell counts to estimate cell concentration ranges of 10^6^ to 10^8^ cells/ml in hot spring environments elsewhere [66].

Several studies have identified significant negative correlations between hot spring temperature and microbial alpha diversity (using Shannon indices) [12–15]. We instead identified a weak, non-significant, negative correlation (R = −0.17, *p* = 0.13; Fig. 2a and S5b), despite significant changes in beta-diversity and inferred biomass. Analysis of TVZ hot spring water (*n* = 925) also showed no association between temperature and alpha diversity, except at >70 °C [6]. However, we found that among features associated with individual hot springs, microbial diversity of lower temperature geothermally-influenced streams (18.3–33.4°C) was significantly higher than in vents and outflows (Figure S6). While the effect of temperature cannot be excluded, this difference could be due to higher reported nutrient (i.e., ammonia, nitrate, and phosphorus) concentrations in the streams [67], supporting a greater diversity of microorganisms. In contrast, results overall indicated a significant positive correlation between pH (range 2.0–7.5) and Shannon diversity (R = 0.28, *p* = 0.01; Fig. 2b), consistent with other TVZ hot spring studies [6, 15]. Although these studies show diversity increases with pH (up to at least pH 9.5), the growth of individual isolates from geothermal environments has been demonstrated across wide pH ranges (e.g., pHs of 1.0–6.0 for *Acidianus brierleyi* and 5.6–10.0 for *Anoxybacillus kamchatkensis*) [68, 69], indicating broad tolerances.

### Predominance of a few microbial taxa across physico-chemically different hot springs

**Fig. 3:**
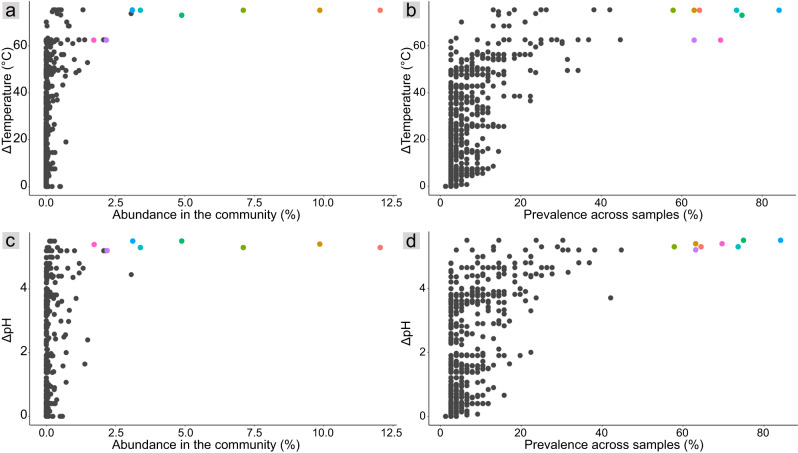
ASV distributions across temperature and pH ranges. **a**, **b** ASV relative abundance (summed across samples) and prevalence versus temperature range. **c**, **d** ASV relative abundance and prevalence versus pH range. Colored dots represent eight ASVs that are highly abundant (>1% abundance in the community) and prevalent (>50% of samples).

**Fig. 4:**
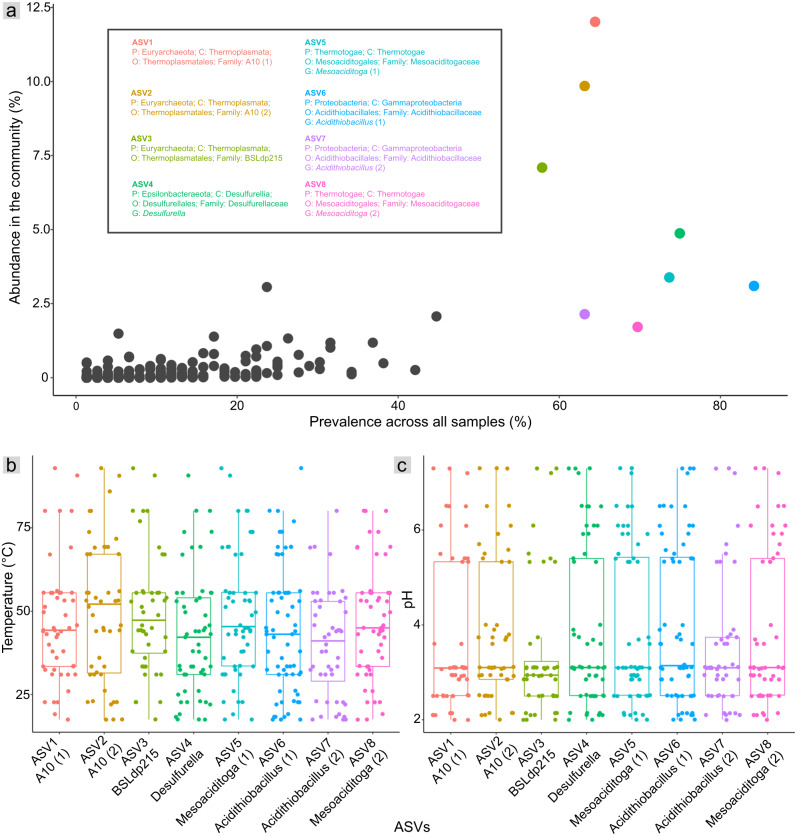
Plots showing ASV abundances and prevalence across temperature and pH ranges. **a** Scatter plot illustrating the overall relative abundance (summed across samples) and prevalence of all ASVs. Colored dots represent eight ASVs that are highly abundant (>1% abundance in the community) and prevalent (>50% of samples). **b**, **c** Temperature and pH ranges where these eight prevalent ASVs were detected. Boxes, internal horizontal lines and whiskers represent upper/lower quartiles, median and minimum/maximum ranges, respectively. The gap between pH 4.1 and 5.3 reflects the lack of hot springs with these pHs in the TVZ, as shown in Fig. 1b.

While microbial alpha diversity and community composition are influenced by pH (Figure S3), some taxa inhabit hot springs with wide-ranging pHs, for example, Sharp et al. [14] reported consistent relative abundances of Proteobacteria, Acidobacteria, Crenarchaeota, and Bacteroidetes across pH gradients. We previously identified a wide occurrence of *Acidithiobacillus* spp. across hot spring sediment and sinter pHs of at least 2.0 to 7.5 [15, 17], likely supported by multiple genes encoding amino acid decarboxylases, K^+^ transporters, Na^+^/H^+^ antiporters, and plasma-membrane proton efflux ATPases [17]. Features such as streamlined genomes and higher predicted proline contents also potentially facilitate the prevalence of TVZ *Acidithiobacillus* across broad hot spring temperature ranges [17]. The wide occurrence of *Acidithiobacillus* across physico-chemically different hot spring sediments and siliceous sinters was achieved by only a few dominant and cosmopolitan species or sub-species, based on an analysis of ASVs and MAGs [17]. To determine how widespread this trend was among other hot spring microorganisms, we examined all bacterial and archaeal variants. Most ASVs were detected at single or narrow ranges of temperature and pH. Specifically, 97.7% of ASVs displayed a temperature range of <10°C, and 98.2% of ASVs had a pH range of <1 pH unit. Nevertheless, some were distributed across large differences, and were found in up to 84% of samples (Fig. 3a–d). We also identified a strong relationship between prevalence and community abundance (R = 0.64, *p* = 2.2 × 10^−16^), with eight of the ten most abundant ASVs overall (comprising up to 12% of the overall community) being the most prevalent (Fig. 4a).

**Fig. 5:**
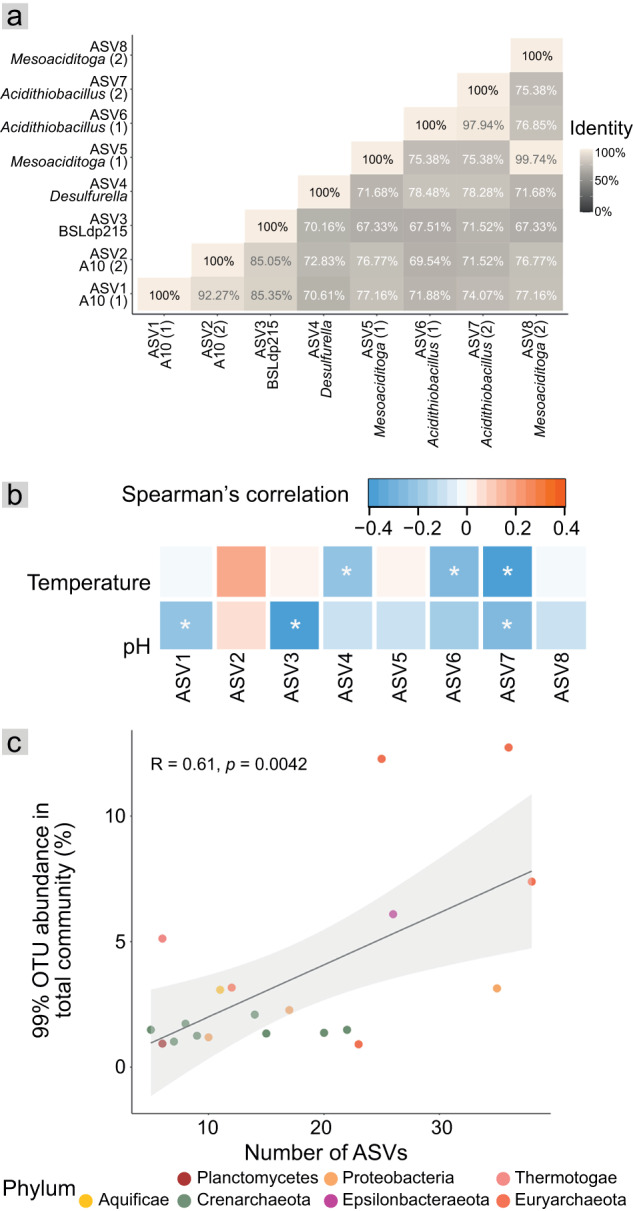
Comparisons of dominant and prevalent variants. **a** Pairwise 16S rRNA gene sequence identities (%) among the eight abundant and prevalent ASVs. **b** Heatmap of Spearman’s correlations between ASV relative abundance and hot spring temperature or pH. Asterisks indicate significant correlations (*p* < 0.05). **c** Plot comparing ASV numbers and OTU relative abundance. The linear trend line shows a significantly positive correlation between the top 20 most abundant OTUs in the total community and number of ASVs observed per OTU. Test = Pearson’s correlation coefficients. Shaded areas represent 95% confidence intervals.

A small fraction of ASVs comprised most of the overall community. In total, 280 ASVs (1.2%) comprised 90% of community abundance. Of these, the eight most prevalent (in >50% of samples) were distributed across temperatures from 17.5 °C and up to 92.9 °C and pHs from 2.0 and up to 7.5 (Fig. 4a–c and S7a). These eight ASVs accounted for 44% of hot spring community abundance and spanned four phyla (pairwise sequence identities 67.3% to 99.7%, Fig. 5a) that are commonly detected in hot spring environments in New Zealand [6, 15] and elsewhere [12, 14]. Three ASVs were Euryarchaeota/Thermoplasmatota (two A10 and one BSLdp215), two each were Proteobacteria (*Acidithiobacillus*) and Thermotogae/Thermotogota (*Mesoaciditoga*), and one was from Epsilonbacteraeota/Campylobacterota (*Desulfurella*). Consistent with their wide hot spring distributions, none showed strong correlations between their relative abundances and either temperature or pH (*r*_*s*_ ≤ 0.4, *p* < 0.05), and three (Thermoplasmatales ASV2 and *Mesoaciditoga* ASVs 5 and 8) showed no significant correlations with either variable (Fig. 5b). All four lineages are well-documented in hot spring settings. For example, *Acidithiobacillus* is also prevalent in TVZ hot spring fluids [6], and has been found in hot spring fluids [46, 70] and sediment [71] elsewhere. At least two species of *Desulfurella* were first discovered from a sulfuric hot spring environment (Kamchatka, Russia) [72, 73]. Similarly, *Mesoaciditogaceae* has been isolated from acidic hot spring water, whereas members of Thermoplasmatota were among the first taxa recovered from hot spring environments [19, 74].

Environments tend to select for phylogenetically related microorganisms based on shared traits [75, 76]. Accordingly, six out of eight of the prevalent and abundant hot spring ASVs we identified were represented by just three taxonomic families or genera (Thermoplasmatales family A10, *Mesoaciditoga*, *Acidithiobacillus*; Fig. 5a) - i.e., there were two-to-three ASVs from each lineage. Results also indicated that rarer close relatives of these variants (those sharing >99% 16S rRNA gene amplicon similarity) were numerous (Supplementary Information and Tables S2-3), likely reflecting environmental selection based on shared traits [75, 76]. ASV numbers were significantly positively correlated with the summed relative abundance of close relatives (OTUs, Fig. 5c and S7b), indicating higher strain or species level diversity among successful hot spring taxa. A similar trend has also been found in communities along oxic-hypoxic gradients of deep lakes [77].

Endemism is suggested to be a feature of hot springs separated by large-scale geographic distances (e.g., different continents) due to dispersal limitations [78, 79]. In this study, the wide distribution of eight abundant microbial variants, up to 65 kilometers apart, and spread across a broad spectrum of temperatures and pHs, suggests cosmopolitanism and not niche differentiation, at least at the 16S rRNA amplicon level of resolution. However, it remains to be determined whether these variants are abundant and prevalent in hot springs outside of the TVZ. Cosmopolitanism of variants has been observed in various other environments. For example, some *Vibrio* oligotypes have both host-associated and free-living lifestyles [80]. ASVs of certain methane-oxidizing bacteria in eutrophic lakes also have been observed across both oxygen-deficient and methane-deficient conditions [81]. Different taxonomic resolution thresholds have been defined based on 16S rRNA genes to determine the cosmopolitanism of closely-related microbial taxa. For example, Ward et al. (2017) allowed 4-nucleotide variation in single ‘sub-OTUs’ [82], while a minute gap of dissimilarity is allowed in single oligotypes (99.2–99.8% identity or 0.2–0.8% sequence variation) [83, 84], and ASVs employ 100% identity [85]. Regardless, observations of dominant sequence variants based on full or partial 16S rRNA gene sequences do not preclude strain-level variation not captured by differences in 16S rRNA genes, which more typically enables genus and, in some cases, species level discrimination based on the full gene [86–88].

### Population structure underpinning the distribution of cosmopolitan variants

**Fig. 6:**
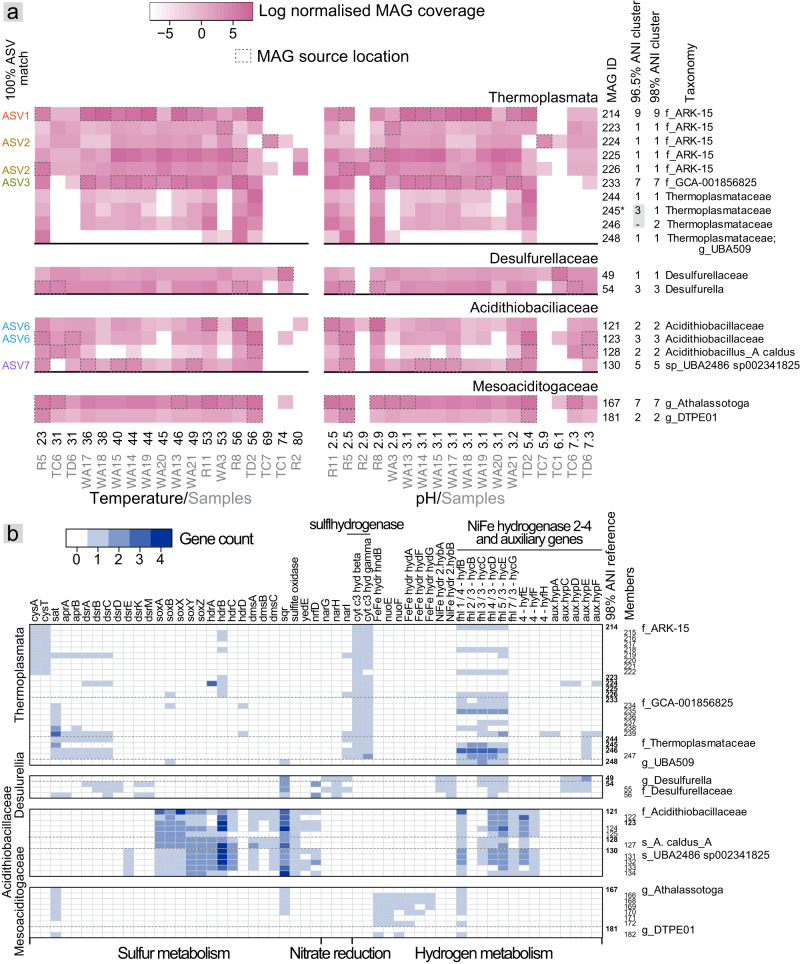
Plots showing the distribution and S/N/H-related metabolic potential of MAGs affiliated with ASV-identified cosmopolitan lineages (Thermoplasmata, Desulfurellia, Acidithiobacillales and Mesoaciditogaceae). **a** Heat maps showing the log relative genome abundance per site based on read mapping. Abundances were normalized to library size, and are included where the summed length of mapped reads equated to least 5% of each genome (72 Kbp to 6 Gbp). Samples are ordered by temperature (left plot), or pH (right plot), and sample conditions and locations are indicated below the x-axes. White dashed boxes indicate samples from which a genome was recovered. MAGs shown are representatives following dereplication at 98% ANI, and MAG cluster sizes based on 98% and 95% ANI thresholds are shown on the right, along with GTDB based taxonomy. Asterisks indicate references for ≥95% ANI clusters. Cosmopolitan ASV sequence matches are shown where 100% identical to a MAG-derived 16S rRNA gene sequence. **b** Heat maps showing gene copy numbers present (maximum = four) per MAG associated with sulfur metabolism (oxidation/reduction), energy-generating nitrogen-cycling processes (only nitrate reduction identified for the MAGs shown), and hydrogen metabolism (production/consumption). hydr hydrogenase, cyt cytochrome, aux auxiliary, fhl formate hydrogenlyase.

To explore the population structures underpinning variant cosmopolitanism, we selected MAGs associated with the eight ASV lineages. Overall, there were 198 MAGs (75-100% complete with <5% contamination) that spanned 35 phyla from 18 hot spring samples (17.5–92.9°C and pH 2.0–7.5). Of these, 50 MAGs were classified as Mesoaciditogaceae, Desulfurellaceae, Acidithiobacillaceae and three Thermoplasmata families (Table S4), and are proposed here to represent 17 unique species based on average nucleotide identities (ANI’s) of ≥96.5% (alignable fractions of 43-96%) [89] (Table S5). Almost half contained 16S rRNA gene sequences, enabling sequence-based comparisons between cosmopolitan ASVs and 20 Thermoplasmata and three Acidithiobacillaceae MAGs (Fig. 6a and Table S4). ASVs 1 and 2 were 100% identical to 16S gene sequences from eight Thermoplasmata family ARK-15 MAGs (NCBI strain identifier A10-Griffin-MG), while ASV3 was identical to six GCA-001856825 family sequences. ASVs 6 and 7 were identical to 16S genes from two MAGs designated as Acidithiobacillaceae (ASV 6) and one Acidithiobacillaceae UBA2486 sp002341825 (ASV 7). Genomes sharing as little as 85% ANI or less can share identical 16S rRNA gene sequences [90], and microorganisms with identical 16S rRNA gene sequences, but divergent genomes, are known to occupy distinct niches [91]. In this study, ASVs 1 and 3 matched with the V4-V5 hypervariable regions of six MAGs each that shared strain-level similarity (≥99% and >98% ANI, respectively). In contrast, ASVs 2 and 6 matched to regions from MAGs with ANIs below the species delineation threshold (91% and 95% ANI), suggesting genus level resolution. Results therefore indicated that these ASVs encompassed collections of strains and distinct species.

To determine the spatial distributions of the 50 MAGs, including those matched to cosmopolitan ASVs, the MAGs were clustered at 98% ANI. Dereplicating populations at this similarity yields a negligible 0.6% chance of indiscriminate mapping (ANI proportion to the power of read length, 0.98^250^) [92]. The resulting 18 representative MAGs had cluster sizes of one to nine, reflecting the recovery of highly similar genomes from multiple hot spring locations (Fig. 6a). Genomes with such high similarity have been termed ‘sequence-discrete’ populations [93–95]. Sequence-discrete populations share >95% nucleotide similarity, are separated from other populations by a genetic discontinuity, and are by inference conspecific [89]. They are more likely to share environmental niches than distinct populations (or species) [94, 95]. Here, genome coverages of representative MAG populations were determined by read mapping. Results indicated the presence of sub-species populations, associated with each of the four archaeal and bacterial lineages, that had broad physicochemical distributions, extending results reported previously for *Acidithiobacillus* [17]. At least one population from each phyla was present in the majority of sites from which metagenomes were derived (16 to 17 sites), which spanned temperatures from 23 to 74 °C or 80 °C and pHs from 2.5 to 7.3 (Fig. 6a, Table S6). Related populations, within and the same families, tended to share broadly similar spatial distribution patterns. However, some differentiation in spatial niches was evident, such as the relatively high abundances of ASV2-associated ARK-15 MAGs 224-226 at 69–80 °C. Striking differences were also evident between Thermoplasmata families - Thermoplasmataceae were all absent (or lower in abundance) at the higher pHs.

### Metabolic traits of cosmopolitan lineages

Differentiation in functional genes as part of the accessory genome is believed to drive niche differentiation among closely-related taxa [96]. For example, key genes for osmolyte uptake are present in the marine subclades/ecotypes of the well-documented alphaproteobacterial bacterioplankton SAR11, but absent in freshwater SAR11 [97]. Similarly, the different responses to light intensity of *Prochlorococcus* ecotypes are likely due to differences in number of putative high-light-inducible proteins [98]. To determine differences in metabolic potential within and between populations we examined Mesoaciditogaceae, Desulfurellaceae, Acidithiobacillaceae, and Thermoplasmata genome annotations for genes involved in sulfur, nitrogen and hydrogen metabolism. Taxa within the four phyla are known for distinct metabolisms. *Desulfurella* and *Acidithiobacillus* are known for sulfur metabolism (reduction and oxidation, respectively), along with iron oxidation by *Acidithiobacillus* species [72], which might explain the prevalence of both genera in the sulfur-rich TVZ hot springs (http://1000springs.org.nz). *Thermotoga* are known as strictly anaerobic fermenters and Mesoaciditogaceae have been shown to reduce thiosulfate and iron [74, 99], while species of Thermoplasmata engage in heterotrophy, methanogenesis or iron oxidation [100, 101]. However, of the TVZ hot spring taxa, all had genes with similarity to those encoding hydrogenases - either FeFe (the Mesoaciditogaceae) or NiFe (Fig. 6b). Mechanisms for sulfur metabolism were ubiquitous, and predictably most numerous in Acidithiobacillaceae and scarcest in Mesoaciditogaceae.

Genomes within each of the four phyla shared characteristic traits (Fig. 6b). For example, all 12 *Acidithiobacillus* MAGs contained *soxABXYZ*, sulfide-quinone reductase (*sqr*), and sulfite oxidase genes. Almost all Thermoplasmata MAGs (24 of 25) contained genes homologous to *asrAB* anaerobic sulfite reductase (analogous to sulfhydrogenase, cytochrome c3 hydrogenase). These were co-located with an archaeal-type formate dehydrogenase alpha subunit with a molybdopterin oxidoreductase 4Fe-4S domain (Table S7), which potentially substitutes for siroheme-binding AsrC oxidoreductase. Asr couples sulfite reduction to hydrogen sulfide with electron acceptors, including NADH, H_2_, and formate [102]. Energy yielding nitrogen cycling mechanisms were largely absent. However, all four Desulfurellaceae MAGs contained *nar* respiratory nitrate reductase genes, and other spatially-localized Campylobacterota relatives present contained periplasmic *nap* nitrate reductase genes (Table S7) [103], indicating that diverse members of this phylum conserve energy via nitrate reduction, albeit via different mechanisms.

Despite shared traits overall, some populations could be delineated from relatives by consistent differences in gene presence or copy numbers. For example, all five Acidithiobacillaceae UBA2486 sp002341825 MAGs were distinguished from other Acidithiobacillaceae by the presence of *dsrE* genes (involved in sulfur transfer) [104], and only a single copy of the *soxABX* sulfur oxidation genes [105]. Comparably, among the Thermoplasmata, only the nine ARK-15 genomes conspecific with MAG 214 had syntenous *cysAT* sulfur transport genes annotated (Fig. 6b) [106], suggesting other Thermoplasmata relied on an alternative mechanism for importing thiosulfate/sulfate. The sulfate adenylyltransferase gene, *sat*, involved in converting sulfur to adenosine 5′-phosphosulfate in the Dsr pathway [107], was instead almost exclusive to other Thermoplasmata families (GCA-001856825 and Thermoplasmataceae).

Some traits appeared to be MAG-specific. In particular, complete sets of adenylyl-sulfate reductase *aprAB* and dissimilatory sulfite reductase *dsrABC* genes were found in a handful of distantly related Thermoplasmata MAGs across all three families. Likewise, syntenous clusters of formate hydrogenlyase (*fhl* subunits 1–5) genes that annotations suggest encode a bidirectional NiFe hydrogenase [108], were present in a small number of ARK-15 MAGs. Although genome incompleteness likely accounts for some differences observed, the complete absence of these genes in a several MAGs, estimated to be 95–99% complete (Table S4) points to strain-level differences in sulfur and hydrogen metabolism.

### Single-nucleotide polymorphisms differentiate populations of Thermoplasmatales

**Fig. 7:**
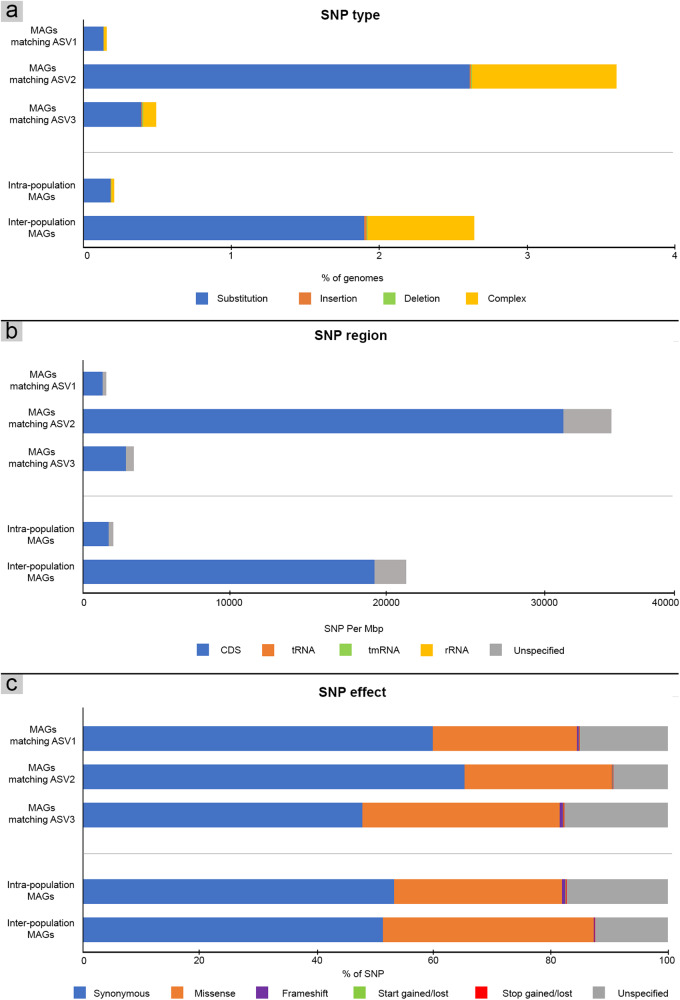
Single-Nucleotide Polymorphisms (SNPs) of MAGs that yielded 16S rRNA gene sequences 100% matched with ASV1, ASV2, and ASV3 of Thermoplasmatales (from Table S5), and SNP rate of members within populations (>99% identity) and between populations (<99% identity). **a** Types of SNP point mutations including substitutions, insertions, and deletions, and complex (i.e., multiple points/combined mutations of substitutions and indels). **b** Genomic regions where SNPs were detected. SNPs identified as ‘unspecified’ were excluded, specifically 200–700 SNPs for intrapopulation and 100-3,200 SNPs for interpopulation MAGs. **c** The effects to CDS by SNPs.

To further explore the genomic diversity among Thermoplasmatales populations, which contained the largest number of genomes and the greatest number of matches to cosmopolitan ASVs, we performed SNP analysis. Results show that SNPs — including indels, non/synonymous point mutations, and complex or multiple/combined point mutations and indels — within a population accounted for an average of 0.2 ± 0.1% of the genomes, whereas the rates between populations rose to 2.6 ± 0.9% (Fig. 7a). Of these SNPs, more than 80% were located in CDS regions (1730 ± 1145 SNPs per Mbp for intra-population and 19,884 ± 12,918 SNPs per Mbp for inter-population). In contrast, only 0–0.03% of SNPs were detected in non-coding rRNA gene regions (0–0.8 SNPs per Mbp for both intra/inter-populations) (Fig. 7b), as expected for the highly conserved nature of 16S rRNA genes. Similarly, *Leptospirillum* and *Ferroplasma* populations, isolated from acid mine drainage, showed no SNPs in their 16S or 23S rRNA gene sequences, while the average SNP rates of their whole genomes were 0.08% and 2.2%, respectively [109]. In addition, we found that about half of SNPs (47.8–66.2%) were synonymous and are not predicted to alter encoded amino acids (Fig. 7c), whereas approximately a quarter were missense SNPs, along with a small number of frameshifts and stop/start codon disruptions (25.1–34.5%) that encode distinct amino acids and may represent phenotypic differences. For example, functional shifts caused by SNPs, which are common among pathogenic microorganisms (e.g., *Staphylococcus aureus*), have been associated with increases in virulence and antibiotic resistance [110]. Comparable to our results, one-third of SNPs detected in *Leptospirillum* and *Ferroplasma* populations in the acid mine drainage study were suggested to cause changes at the protein-coding level [109]. Taken together, our results imply that the cosmopolitanism observed among hot spring ASVs encompasses genomic variation resulting from intra- and inter-population point mutations (as illustrated with Thermoplasmatales), along with differences in metabolic gene composition.

## Conclusions

This study reports the influence of temperature and pH on the relative abundance, diversity, and prevalence of bacteria and archaea in hot spring sediments. We found that bacteria and archaea shared similar abundances in the overall hot spring microbial communities, despite a slight proportional increase in archaea with higher temperatures. The dominant phyla overall were a mixture of bacteria (Proteobacteria) and archaea (Euryarchaeota and Crenarchaeota). Higher microbial diversity was associated with an increase in pH from 2.0 to 7.5, while microbial cell concentrations, as inferred from 16S rRNA gene copies, were primarily influenced by and positively correlated with temperature. Although differences in microbial composition were driven by temperature and pH, we identified eight phylogenetically diverse bacterial and archaeal variants (based on ASVs) that were found in up to 84% of the hot spring communities and accounted for 44% of the relative abundance. These variants, belonging to Thermoplasmatales, *Desulfurella*, *Mesoaciditoga*, and *Acidithiobacillus*, were present across geographically distant hot spring sites with wide-ranging temperatures and pHs, illustrating the lack of constraint of temperature and pH on their distributions. Amplicon results suggest bacterial and archaeal cosmopolitanism may be a common feature of hot spring environments. Metagenomic results indicated the presence of strain-level (sub-species) populations associated with each of these four lineages that had broad spatial and physicochemical ranges. In general, diverse members of each lineage (different families or genera) shared common metabolic traits, although variations in mechanisms for sulfur, nitrogen and hydrogen metabolism were evident both between and within predicted species. Accordingly, when inspecting nucleotide-level difference among the numerous Thermoplasmata MAGs recovered, we found that both intra-species, and to a greater extent, inter-species populations were differentiated by a mixture of synonymous and non-synonymous SNPs, indicating differences in amino acid coding and potential differences in protein function. Results suggest that multiple prokaryotic lineages, including both bacteria and archaea, are successful in colonizing a range of hot spring conditions by harboring diverse population structures and genome-specific metabolic traits.

## Supplementary information

Supplemental information

Data Set 1

## Data Availability

Sequence data can be accessed through NCBI BioProject PRJNA644733.
