# Supplementary material for: Bacterial and archaeal community distributions and cosmopolitanism across physicochemically diverse hot springs
Source: ISME Commun. 2023 Aug 18;3:80. doi: 10.1038/s43705-023-00291-z (PMC10439147; doi:10.1038/s43705-023-00291-z)
Supplement: Supplementary file 1 — Supplemental information [file 43705_2023_291_MOESM1_ESM.pdf]

Supplementary Information *(Methods, Results and Figures)*

Sriaporn et al. Bacterial and archaeal community distributions and cosmopolitanism across physicochemically diverse hot springs

Supplementary Methods

DNA extraction, amplicon and metagenomic sequencing

DNA extractions for 16S rRNA gene amplicons were performed using the DNeasy PowerSoil Pro® Kit (Qiagen, USA) and 0.25-0.35 g of sediment per sample. 16S rRNA gene amplification was performed using 515′F/926R primers (GTGBCAGCMGCCGCGGTAA and CCGYCAATTYMTTTRAGTTT), targeting the V4-5 regions, with Illumina adapters attached [1, 2, 3]. Agencourt AMPure XP magnetic beads (Beckman Coulter) were used to purify PCR products. Barcoded libraries were prepared, and 2 × 250 bp paired-end Illumina MiSeq sequencing was performed with the V2 MiSeq Reagent Kit (by Auckland Genomics, University of Auckland, New Zealand).

For metagenomics, the DNeasy PowerMax Soil Kit® (Qiagen, USA) was used for gDNA extraction from 5-7 g of each of 18 representative sediment samples [4]. DNA was concentrated and cleaned by ethanol precipitation. Metagenomic sequencing was conducted at the Otago Genomics Facility (University of Otago, Dunedin, New Zealand). Thruplex DNA-Seq libraries (Takara Bio USA, Inc, USA) were prepared with 500-600 bp insert sizes and sequenced using the Illumina HiSeq 2500 SBS Kit V4, yielding 2×125 bp reads.

Metagenome assembly, binning and annotation

Paired-end trimmed reads from each sample were assembled separately using SPAdes version 3.11.1 [5]. To determine differential coverage for genome binning, reads were mapped to contigs using Bowtie version 1.2.0 [6]. Contigs were binned using MetaBAT version 2.12.1 [7], MaxBin version 2.2.4 [8], and CONCOCT version 0.4.1 [9], utilizing differential coverage and tetranucleotide frequencies. Representative bin (Metagenomic-assembled Genomes; MAGs) were selected using DAS Tool version 1.1.1 [10], and refined using VizBin with

differential coverage [11]. CheckM version 1.0.12 was used to estimate refined bin completeness and contamination [12].

Gene prediction was performed using Prodigal version 2.6.3 [13]. Predicted coding DNA sequences (CDS) were annotated against the UniRef100 [14], UniProt [15], KEGG [16], Pfam [17], and TIGRFAM [18] databases using USEARCH version 9.0.2132 [19]. Taxonomic classification was assigned based on core marker genes using GTDB-Tk version 0.2.1 [20].

Supplementary Results and Discussion

Bacterial and archaeal relative abundance

While our results show that archaea are at least as abundant as bacteria in acidic to circumneutral pH TVZ hot spring sediments, an extensive study of hot spring water samples in the TVZ showed that the abundance of archaea was much lower than bacteria (6.4% archaeal versus 93.6% bacterial compared with 51.5% versus 48.5% in this study for genera >1% average community abundance [21]). Notably, only 1.9% average abundance was previously identified for Thermoplasmatales (Euryarchaeota) in hot spring environments [21], whereas in this study, we report an average abundance of 32.2 ± 27.7% (37.6% of the overall hot spring community). The range of temperatures and pHs, especially acidic pH which is favorable for Thermoplasmatales, tested were broadly similar to those in this study (i.e. 13.9-100.6°C and pH -0.8-9.7 versus 17.5-92.9°C and pH 2.0-7.5), and therefore cannot account for the difference. The discrepancy may instead be due to differences between water and sediment community compositions [22], or it may be explained by differences in primers used between the studies. In particular, it has been suggested that the previously used V4 primer set (515F/806R), as originally employed for the Earth Microbiome Project [23], exhibits biases toward the archaeal domain, resulting in its underestimation [1, 2, 3], a point also acknowledged by Thompson et al. [23] and Power et al. [21]. The 515′F/926R primer set (V4-V5 hypervariable regions), developed by Hugerth et al. [2], Walters et al. [3], and Parada et al. [1], and used here, improves detection of certain archaeal clades such as Euryarchaeota and Thaumarchaeota – both observed in this study (Figure 1a). The V4-V5 hypervariable region has also been shown to be among the most reliable regions for approximating the taxonomic resolution of the full-length 16S rRNA gene [24, 25].

Predominance of a few microbial variants across physico-chemically different hot springs

To examine the extent that the eight most prevalent and abundant ASVs, and other, predominant ASVs represent populations of closely related taxa, we clustered all ASVs based on an identity threshold of 99%. This threshold was selected because we previously found that *Acidithiobacillus* ASVs (ASV6 and ASV7 in Figure 5a) clustered together at 98% sequence identity (they are 97.94% identical), although associated MAGs formed phylogenetically distinct clades when compared using a set of protein-coding core genes (GTDB-Tk bacterial marker gene set, *Acidithiobacillus* TVZ_G3 and TVZ_G2 in Sriaporn et al. [4]). In this analysis, 19,196 operational taxonomic units (OTUs) were generated. ASVs belonging to the 20 most abundant OTUs, accounting for 70.1% of overall community abundance, were examined, and included the previously identified eight prevalent variants. Of the prevalent variants, ASVs 5 and 8 (*Mesoaciditoga*) were highly similar, sharing 99.74% sequence identity, and clustered together.

Among the 20 most abundant OTUs, the number of variants per OTU was 17.5 on average, with a maximum of 38 variants per OTU (BLSdp215 group from Thermoplasmatales), and a minimum of 5 variants per OTU (*Thermodiscus*) (Figure S7b, Tables S2), indicating high variant diversity. Rarefaction curves representing ASV diversity indicated saturation was being approached, but was not yet achieved in most sample sites (Figure S8), which suggests that additional sequencing depth could yield more rare variants. Despite the relationship between OTU diversity and community abundance (Figure 5c), we typically found that only one to two variants (e.g., the eight prevalent variants) dominated each OTU, accounting for 70.0-99.9% relative abundance of the respective OTUs, while other variants were rare (Figure S7b, Table S2).

References

1. Parada AE, Needham DM, Fuhrman JA. Every base matters: assessing small subunit rRNA primers for marine microbiomes with mock communities, time series and global field samples. Environmental Microbiology. 2016;18(5):1403-14.

2. Hugerth L, W., Wefer H, A., Lundin S, Jakobsson H, E., Lindberg M, Rodin S, et al. DegePrime, a program for degenerate primer design for broad-taxonomic-range PCR in microbial ecology studies. Applied and Environmental Microbiology. 2014;80(16):5116-23.

3. Walters W, Hyde Embriette R, Berg-Lyons D, Ackermann G, Humphrey G, Parada A, et al. Improved bacterial 16S rRNA gene (V4 and V4-5) and fungal internal transcribed spacer marker gene primers for microbial community surveys. mSystems. 2015;1(1):e00009-15.

4. Sriaporn C, Campbell KA, Van Kranendonk MJ, Handley KM. Genomic adaptations enabling Acidithiobacillus distribution across wide-ranging hot spring temperatures and pHs. Microbiome. 2021;9(1):135.

5. Bankevich A, Nurk S, Antipov D, Gurevich AA, Dvorkin M, Kulikov AS, et al. SPAdes: a new genome assembly algorithm and its applications to single-cell sequencing. Journal of Computational Biology. 2012;19(5):455-77.

6. Langmead B. Aligning short sequencing reads with Bowtie. Current Protocols in Bioinformatics. 2010;32(1):11.7.1-11.7.4.

7. Kang DD, Froula J, Egan R, Wang Z. MetaBAT, an efficient tool for accurately reconstructing single genomes from complex microbial communities. PeerJ. 2015;3:e1165.

8. Wu Y-W, Tang Y-H, Tringe SG, Simmons BA, Singer SW. MaxBin: an automated binning method to recover individual genomes from metagenomes using an expectation-maximization algorithm. Microbiome. 2014;2(1):26.

9. Alneberg J, Bjarnason BS, de Bruijn I, Schirmer M, Quick J, Ijaz UZ, et al. Binning metagenomic contigs by coverage and composition. Nature Methods. 2014;11(11):1144-6.

10. Sieber CMK, Probst AJ, Sharrar A, Thomas BC, Hess M, Tringe SG, et al. Recovery of genomes from metagenomes via a dereplication, aggregation and scoring strategy. Nature Microbiology. 2018;3(7):836-43.

11. Laczny CC, Sternal T, Plugaru V, Gawron P, Atashpendar A, Margossian HH, et al. VizBin - an application for reference-independent visualization and human-augmented binning of metagenomic data. Microbiome. 2015;3(1):1.

12. Parks DH, Imelfort M, Skennerton CT, Hugenholtz P, Tyson GW. CheckM: assessing the quality of microbial genomes recovered from isolates, single cells, and metagenomes. Genome Research. 2015;25(7):1043-55.

13. Hyatt D, Chen G-L, LoCascio PF, Land ML, Larimer FW, Hauser LJ. Prodigal: prokaryotic gene recognition and translation initiation site identification. BMC Bioinformatics. 2010;11(1):119.

14. Suzek BE, Huang H, McGarvey P, Mazumder R, Wu CH. UniRef: comprehensive and non-redundant UniProt reference clusters. Bioinformatics. 2007;23(10):1282-8.

15. Wu CH, Apweiler R, Bairoch A, Natale DA, Barker WC, Boeckmann B, et al. The Universal Protein Resource (UniProt): an expanding universe of protein information. Nucleic Acids Research. 2006;34(suppl_1):D187-D91.

16. Kanehisa M, Goto S. KEGG: Kyoto Encyclopedia of Genes and Genomes. Nucleic Acids Research. 2000;28(1):27-30.

17. Finn RD, Bateman A, Clements J, Coggill P, Eberhardt RY, Eddy SR, et al. Pfam: the protein families database. Nucleic Acids Research. 2014;42(D1):D222-D30.

18. Haft DH, Selengut JD, White O. The TIGRFAMs database of protein families. Nucleic Acids Research. 2003;31(1):371-3.

19. Edgar RC. Search and clustering orders of magnitude faster than BLAST. Bioinformatics. 2010;26(19):2460-1.

20. Chaumeil P-A, Mussig AJ, Hugenholtz P, Parks DH. GTDB-Tk: a toolkit to classify genomes with the Genome Taxonomy Database. Bioinformatics. 2020;36(6):1925-7.

21. Power JF, Carere CR, Lee CK, Wakerley GLJ, Evans DW, Button M, et al. Microbial biogeography of 925 geothermal springs in New Zealand. Nature Communications. 2018;9(1):2876.

22. Colman DR, Feyhl-Buska J, Robinson KJ, Fecteau KM, Xu H, Shock EL, et al. Ecological differentiation in planktonic and sediment-associated chemotrophic microbial populations in Yellowstone hot springs. FEMS Microbiology Ecology. 2016;92(9):fiw137.

23. Thompson LR, Sanders JG, McDonald D, Amir A, Ladau J, Locey KJ, et al. A communal catalogue reveals Earth's multiscale microbial diversity. Nature. 2017;551(7681):457-63.

24. Chaudhary N, Sharma AK, Agarwal P, Gupta A, Sharma VK. 16S classifier: a tool for fast and accurate taxonomic classification of 16S rRNA hypervariable regions in metagenomic datasets. PLOS One. 2015;10(2):e0116106.

25. Yang B, Wang Y, Qian P-Y. Sensitivity and correlation of hypervariable regions in 16S rRNA genes in phylogenetic analysis. BMC Bioinformatics. 2016;17(1):135.

Supplementary Figures

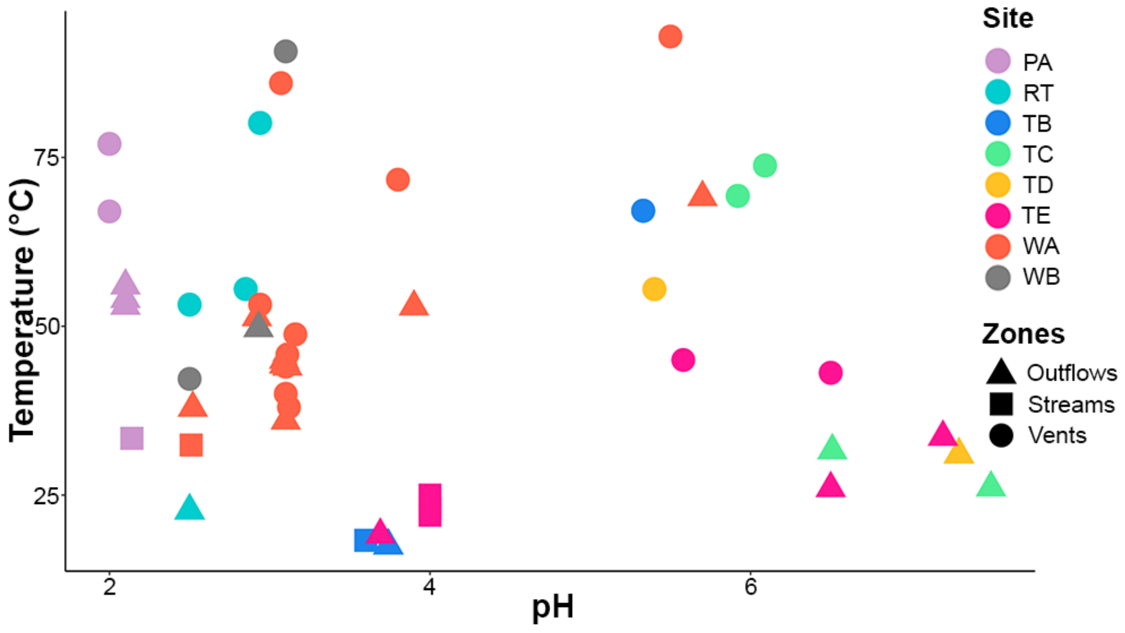

Figure S1 A scatterplot showing temperature (°C) and pH distributions studied hot spring samples.

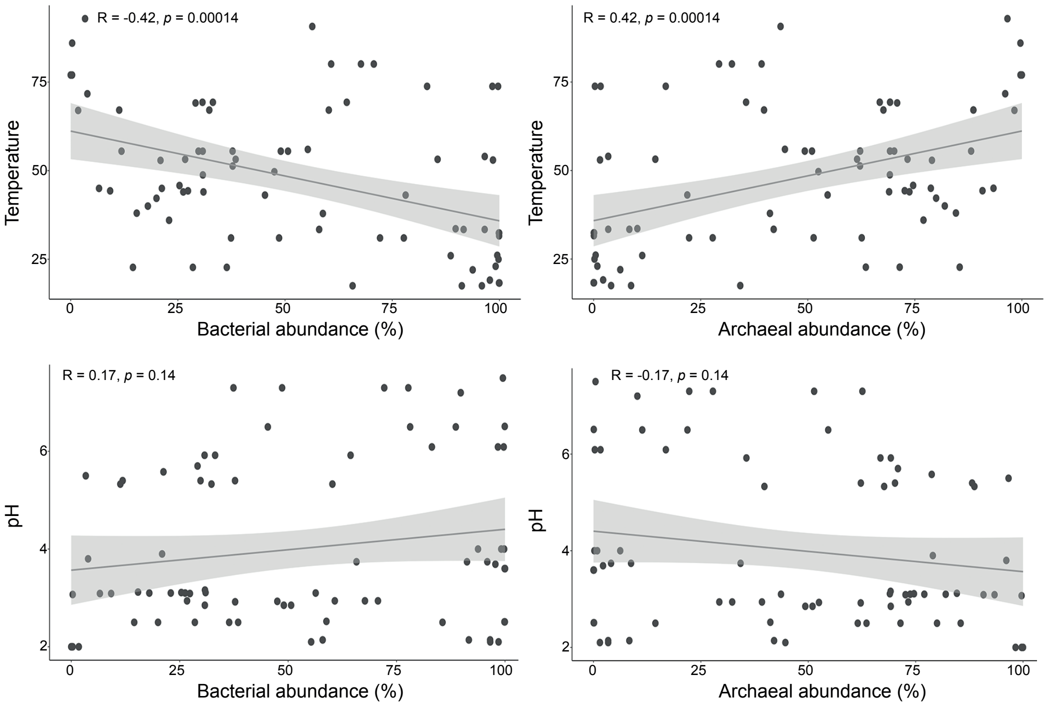

Figure S2 Correlations between physicochemistry and the relative abundances of bacteria or archaea. Trend lines indicate linear correlations that depict a) a significantly negative association between bacterial abundance (%) and temperature (°C), and b) a significantly positive association between archaeal abundance (%) and temperature (°C). c-d) Correlations with pH were not significant. Test = Pearson's correlation coefficients. Shaded areas represent 95% confidence intervals.

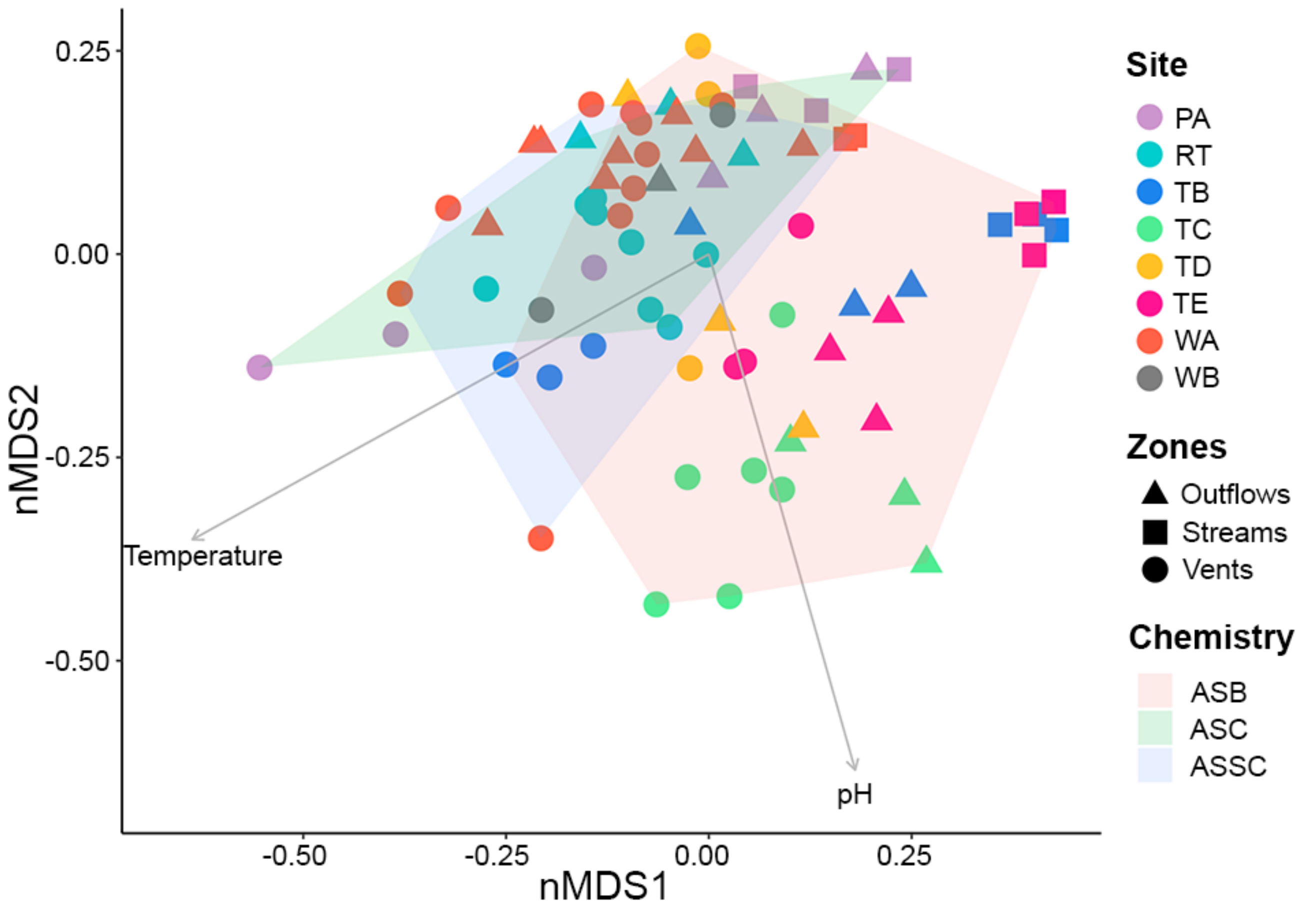

Figure S3 Non-metric multidimensional scaling (nMDS) plot based on hot spring microbial community Bray-Curtis dissimilarities. Vectors indicate directions where temperature and pH have effects.

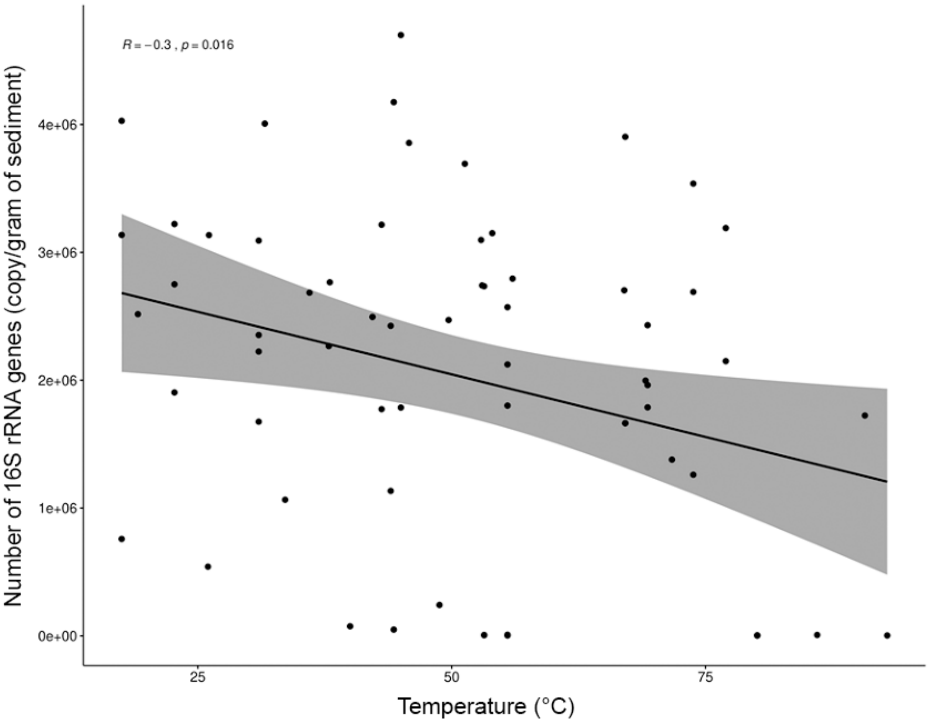

Figure S4 Plot comparing water temperature with 16S rRNA gene copy numbers. The linear trend line shows a significantly negative correlation between number of gene copies and temperature, excluding geothermal-influenced stream samples. Shaded areas represent 95% confidence intervals.

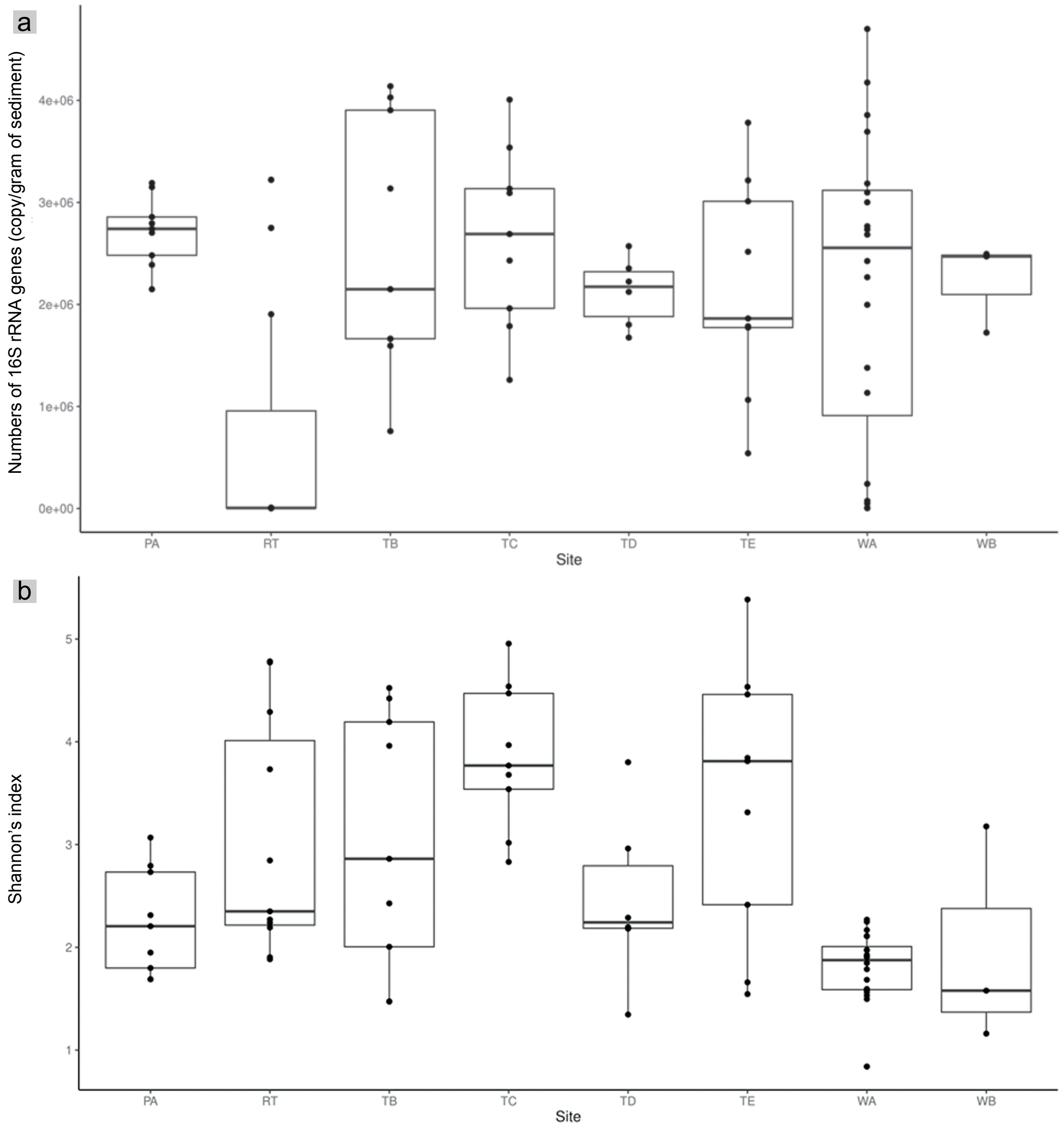

Figure S5 Box plots of inferred microbial biomass and alpha diversity across hot spring sampling sites. (a) 16S rRNA gene concentrations (quantified by ddPCR) at each hot spring site. (b) Shannon's indices of microbial communities. (a-b) Black dots represent individual samples. Boxes, internal horizontal lines and whiskers represent upper/lower quartiles, median and minimum/maximum ranges, respectively. Acronyms represent sites following Table S1.

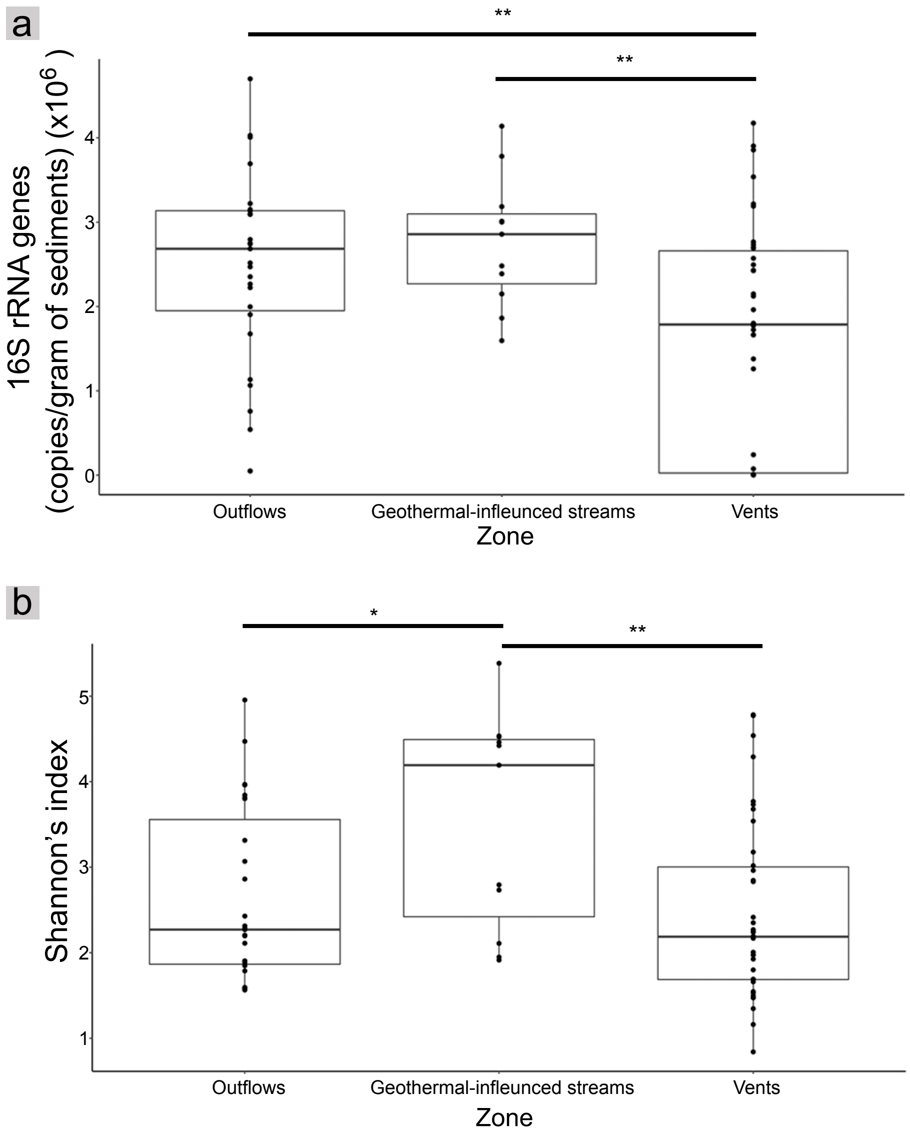

Figure S6 Boxplots showing inferred biomass and alpha diversity for each hot spring zone. a) Numbers of 16S rRNA genes of microbial communities from outflows, geothermal-influenced streams, and vents. Dunn's test was used to calculate the statistical differences: outflows and vents; $p = 0.0054$, geothermal-influenced streams and vents; $p = 0.0082$. (b) Shannon's index of microbial communities from outflows (average temperature = 36.1 ± 13.9°C), geothermal-influenced streams (average temperature = 26.3 ± 6.4°C), and vents (average temperature = 62.1 ± 15.0°C). Dunn's test was used to calculate the statistical differences: outflows and geothermal-influenced streams; $p = 0.02$, geothermal-influenced streams and vents; $p = 0.0048$. Horizontal lines over plots and * symbols represent significant differences in pairwise copy numbers (*, <0.05; **, <0.01). Boxes, internal horizontal lines and whiskers represent upper/lower quartiles, median and minimum/maximum ranges, respectively.

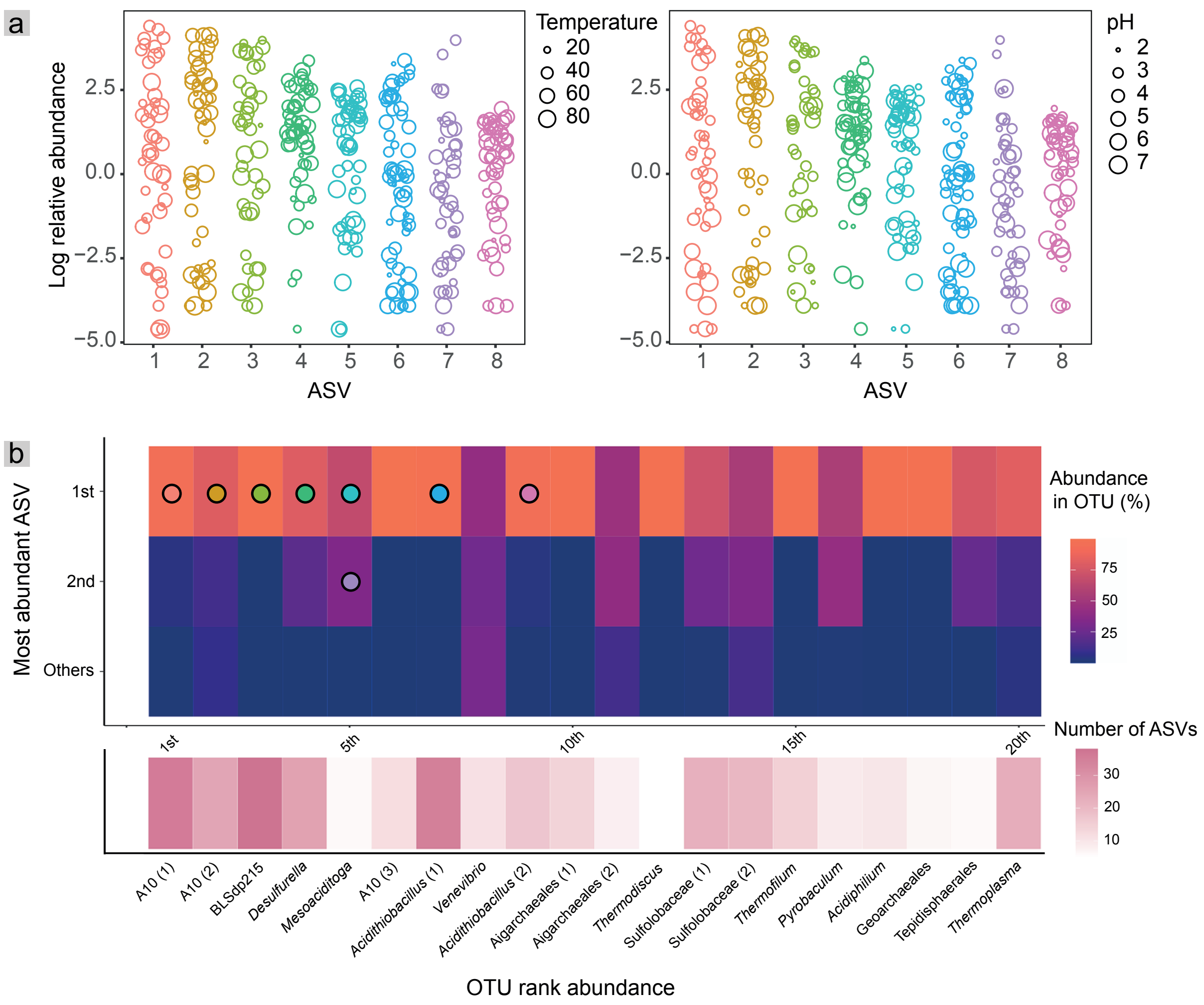

Figure S7 Heatmaps showing the relative abundances of, and relationship between, the top eight ASVs and 20 OTUs. (a) Strip plots showing the log relative abundance of the eight most abundant and prevalent ASVs in relation to sample temperature (left) and pH (right). (b) Plots show the relative abundances (summed across all samples) of the eight ASVs (indicated by coloured circles) and the top 20 most abundant OTUs (upper heatmap), and the number of ASVs identified in the OTUs (lower heatmap).

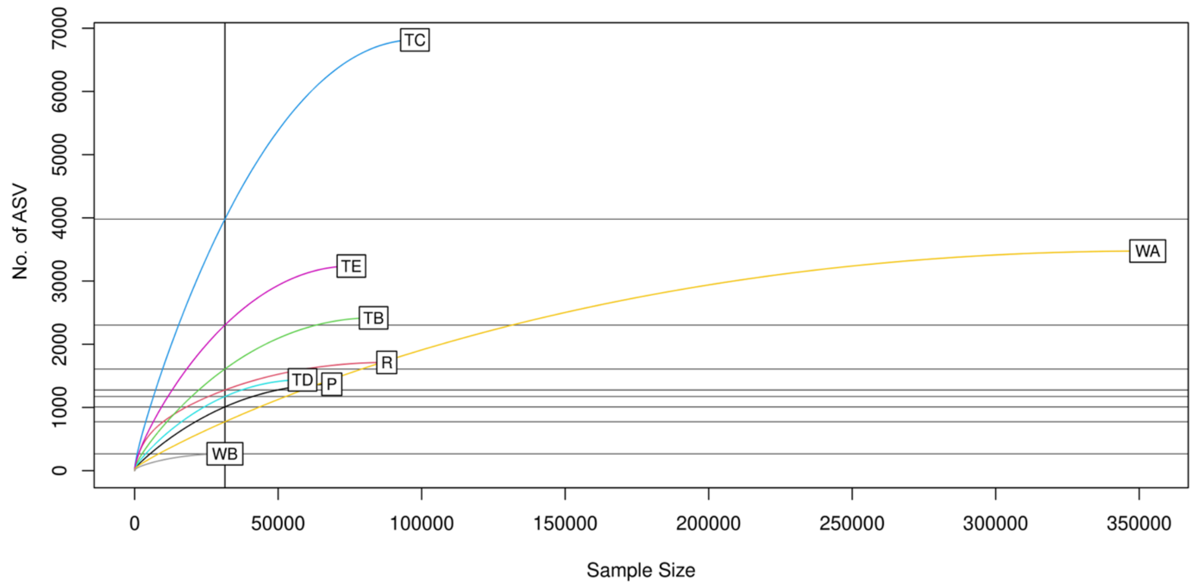

Figure S8 Rarefaction curves showing number of ASVs per site that were obtained from sequencing from each site in this study. Curves were flattening (slopes approaching zeros), although in most cases saturation was not yet achieved, indicating further sequencing would yield additional rare ASVs. Vertical lines = minimum sequence yield. Acronyms represent sites following Table S1.
